# Editorial: Emerging Functions of Septins

**DOI:** 10.3389/fcell.2017.00073

**Published:** 2017-08-24

**Authors:** Manoj B. Menon, Matthias Gaestel

**Affiliations:** Institute for Cell Biochemistry, Hannover Medical School Hannover, Germany

**Keywords:** septin, cytokinesis, cytoskeleton, SEPT, GTPase, signaling

Septins are a family of conserved cytoskeletal GTPases with unique heteropolymerization properties and diverse physiological functions (reviewed in Mostowy and Cossart, 2012). Originally discovered in the budding yeast *Saccharomyces cerevisiae* as factors crucial for the mother-bud separation, septin genes were later identified in almost all eukaryotes except higher plants. Initial studies on septins were limited to their functions in yeast, with a major focus on cell division. However, much information has been gathered about mammalian septins in the present century and septins are gaining importance as a distinct fourth component of the mammalian cytoskeleton. The presence of multiple septin genes with possibly redundant roles and the lack of potent and specific septin inhibitors have hindered functional characterization of the septin cytoskeleton. These difficulties have been effectively tackled by the widespread use of RNAi technologies and at least 10 out of the 13 mouse septin genes have been successfully knocked out, aiding in the identification of several general and isoform-specific physiological functions of septins. This Frontiers Research topic on the emerging functions of septins compiles contributions from the diverse field of septin research dealing with complex issues from yeast cell division to human cancer, from cellular morphogenesis to protein stability and from plant pathogens to human infections.

Historically, the name “septin” originated from their role in “septa” formation in the budding yeast. Septins participate in crucial cell cycle phases in *S. cerevisiae* and the mini-review from Glomb and Gronemeyer summarizes our current understanding on septin functions in this model organism, with a special focus on the subcellular organization and septin assembly. There is a long-standing view that septins could act as signaling scaffolds facilitating localized regulatory events. Perez et al. discuss septins as kinase scaffolds explaining the relevance of these septin associated kinases in the regulation of different cell cycle phases in the budding yeast. They describe the salient features of septin-dependent cell-cycle checkpoints, which determine the precise time of mitotic entry, exit, and cytokinesis. In addition to the role of septin scaffolds in coupling cell morphology to cell cycle decisions, the different signaling events regulating septin assembly are also discussed here. The complexity in the regulatory role for septins in yeast budding is further addressed by McQuilken et al. by monitoring septin reorganization at the bud neck using advanced polarized fluorescence microscopy. By the systematic analysis of multiple mutants lacking septin interacting proteins, they further establish a complex role for kinases in septin remodeling at the bud-neck. While the above mentioned articles focus on the model organism *S. cerevisiae*, Momany and Talbot present a different view on how septins are essential for fungal pathogenesis. Taking examples from human and plant pathogenic fungi they discuss the importance of septin-driven morphological changes as a prerequisite for focusing invasive force in an efficient and directed manner to facilitate pathogenesis.

Septins associate with membranes and can facilitate selective diffusion of components, a property of septin family contributing to processes including neuronal morphogenesis, ciliogenesis, and sperm motility. In a thought-provoking mini review article, Palander et al. discuss the current understanding and possible functions of septins in the biogenesis of cilia and sperm flagella. The research article by Kaplan et al. addresses the functional redundancy of SEPT2 family members in neuronal morphogenesis, concluding that SEPT2, SEPT4, and not SEPT1 can compensate for the loss of SEPT5 in dendritic branching. In the context of membrane association of septins, the mini review by Song et al. deals with the evidences which indicates a role for septins in endo-lysosomal membrane traffic. They discuss the role of septins in vesicular trafficking and autophagy, as indicated by multiple septin-interactors with essential roles in these processes. In a completely different approach, Neubauer and Zieger summarize the current status of the mammalian septin interactome with important additional insights into their previous findings on clinically relevant septin interactors of human platelets and endothelial cells. Septins are described here as key mediators of platelet degranulation and, accordingly, the authors also deliberate on the clinical relevance of the *SEPT5* gene locus in bleeding disorders.

Since septins are crucial player in multiple stages of cell division and septin gene translocations and fusions were detected in leukemia, septins have been implicated in cancer development (Cerveira et al., 2011). While a direct functional role for septin in tumorigenesis has not been established yet, there are several studies linking septins to diverse forms of human cancer, such as the use of *SEPT9* gene methylation as a biomarker for colon cancer (Warren et al., 2011). In a very unique contribution on this subject, Poüs et al. look at the cancer related functions of septins through the prism of their subcellular localization. This alternate view of localization-dependent septin functions in cancer may ignite the interest of researchers from diverse fields to investigate septin functions in tumorigenesis. This article is complemented by a thought-provoking review article of Angelis and Spiliotis who provide a comprehensive view of septin mutations in human cancers. By an approach coupling large scale sequence data analysis and basic septin biochemistry, the authors convince the readers that despite the complexity associated with multiple septin genes and diverse cancers, there are recurring mutations on conserved amino acids across the septin family. Interesting observations on the possible effects of these mutations on septin oligomerization and function are described. Being the most investigated septin gene in the field of cancer, the authors also give due importance to SEPT9, and separately discuss the mutations of the unique N-terminal domain of SEPT9.

The immune-related functions of septins are also often linked to their membrane association and interplay with the cortical actin cytoskeleton. Despite normal development of hematopoietic lineages on pan-septin depletion in the *Sept7* knockout mouse model (Menon et al., 2014), septins regulate lymphocyte migration (Tooley et al., 2009) and associate with macrophage phagosomes (Huang et al., 2008), indicating a clear participation in the immune response. A significant role for septins in host cell entry and invasiveness of intracellular bacterial pathogens in non-phagocytic cells was one of the more recent discoveries in the field (Mostowy et al., 2010). In their mini review Torraca and Mostowy discuss this role of septins in bacterial infection together with its interplay with the autophagy pathway. The diverse methods employed by bacterial pathogens to take advantage of the host cytoskeleton to gain entry into cells and the counter mechanisms in place are nicely described by the authors here. In the context of bacterial pathogenesis, it was recently shown that the intracellular longevity of *Clostridium botulinum* neurotoxin Bont/A arise from their association with septins (Vagin et al., 2014). Vagin and Beenhouwer discuss this concept in their mini review dealing with the role of septins as regulators of protein stability. They broadly look into the role played by septins in regulating the stability of pathogenic toxins as well as other signaling molecules, which could be relevant to cancer as well as immunity.

One of the latest discovered functions of septins concerns its involvement in the store operated calcium entry (SOCE). While an siRNA screen identified SEPT2 family members as mediators of calcium signaling in HeLa cells (Sharma et al., 2013), T cell calcium signaling was not affected in a conditional *Sept7* knockout mouse model (Mujal et al., 2016). Studies in the Drosophila model also suggests an isoform specific role for septins in regulating SOCE (Deb et al., 2016). A perspective article by Deb and Hasan discuss the intricacies associated with these findings and their possible relevance to mammalian neuronal function.

Our understanding on the canonical functions of septins in cell division is constantly being revisited. In addition, the foot print is spreading far and wide as more and more physiologically relevant functions emerge for the septin family proteins. Collectively, the articles in this research topic including original research, perspectives and reviews on diverse aspects of septin research comprehensively summarizes the advances in the field. The contributions also provide ideas of promising directions for future investigations.

## Author contributions

Both authors equally contributed to the drafting and revision of the editorial and approved it for publication.

### Conflict of interest statement

The authors declare that the research was conducted in the absence of any commercial or financial relationships that could be construed as a potential conflict of interest.
